# Usability of a Mobile Phone App Aimed at Adolescents and Young Adults During and After Cancer Treatment: Qualitative Study

**DOI:** 10.2196/15008

**Published:** 2020-01-02

**Authors:** Signe Hanghøj, Kirsten A Boisen, Maiken Hjerming, Abbey Elsbernd, Helle Pappot

**Affiliations:** 1 Center of Adolescent Medicine Department of Paediatrics and Adolescent Medicine The Copenhagen University Hospital, Rigshospitalet Copenhagen Denmark; 2 Department of Hematology Department of Paediatrics and Adolescent Medicine The Copenhagen University Hospital, Rigshospitalet Copenhagen Denmark; 3 University of Kansas School of Medicine Kansas City, KS United States; 4 Department of Oncology The Copenhagen University Hospital, Rigshospitalet Copenhagen Denmark

**Keywords:** AYA, adolescent and young adult, app, cancer, co-creation, mHealth, mobile phone, think-aloud test, usability

## Abstract

**Background:**

Adolescent and young adult (AYA) cancer patients are seldom involved in the process of testing cancer-related apps. As such, knowledge about youth-specific content, functionalities, and design is sparse. As a part of a co-creation process of developing the mobile phone app Kræftværket, AYAs in treatment for cancer and in follow-up participated in a usability think-aloud test of a prototype of the app. Thus, the app was initiated, created, and evaluated by AYAs with cancer experience.

**Objective:**

The aim of this study was to explore the results of a think-aloud test administered to see how the prototype of the app Kræftværket was used by AYAs in treatment for cancer and in follow-up, and to investigate the strengths and weaknesses of the app.

**Methods:**

A total of 20 AYA cancer patients aged 16 to 29 years (n=10 on treatment, n=10 in follow-up) were provided with the first version of the co-created mobile phone app Kræftværket during a 6-week test period (April-May 2018). After the test period, 15 participated in individual usability think-aloud tests. The tests were video-recorded, transcribed verbatim, and analyzed using a thematic analysis approach.

**Results:**

The thematic analysis led to the following themes and subthemes: navigation (subthemes: intuition, features, buttons, home page, profile), visual and graphic design (subthemes: overview, text and colors, photos, videos, YouTube), and usefulness (subthemes: notifications, posts, adding). The analysis identified gender differences in app utilization—female participants seemed to be more familiar with parts of the app. The app seemed to be more relevant to AYAs receiving treatment due to app functions such as tracking symptoms and searching for relevant information. Lack of notifications and incorrect counting of posts were perceived as barriers to using the app.

**Conclusions:**

Usability testing is crucial to meet the needs of the AYA target audience. AYA cancer apps should preferably be relevant, targeted, and unique, and include a tracking function and AYA-produced videos. Notifications and correct marking and ordering of posts are critical to make apps engaging and dynamic. Further research is recommended to evaluate the Kræftværket app with the input of more AYAs.

## Introduction

In Denmark, approximately 500 adolescents and young adults (AYAs) aged 15 to 29 years are diagnosed with cancer each year. Worldwide, there has been an increased focus on AYA cancer patients as a group with special treatment and support needs, addressing problems such as social maturity, identity-forming, health concerns, romantic relationships, friendships, fertility, mood changes, and risk of depression and anxiety [1,2]. Mobile health (mHealth) apps have demonstrated benefits in addressing some of these needs of AYAs, including connecting with peers and health care teams, accessing information, and health care tracking [3-5]. For AYA cancer patients, networking with peers, information seeking, and tracking of symptoms are found to be most relevant at diagnosis onset and during the initial treatment period [6,7], and the motivation to use health apps often decreases over time [8,9]. Differences in the supportive care needs for AYAs in treatment and off treatment are common, particularly regarding information about the disease, treatment, and side effects versus information about risks of recurrence and potential late effects [10].

Internationally, several apps have been developed for AYAs with cancer, but AYAs have rarely been involved in the development process [11,12] despite research indicating that user involvement in the development of mHealth solutions is necessary to ensure relevant content and functionality [13,14]. Moreover, it is important to involve AYAs during app development and evaluation because they are a target group highly familiar with mobile technology [15], and they are discerning and critical users of digital health technologies [16]. Research suggests that patient-oriented apps may strengthen patients’ empowerment [17] and that apps are effective tools for enhancing self-management in both younger and older patients [18,19]. Additionally, digital health intervention apps have been shown to address unmet psychosocial and health information needs of AYA [20]. Research also points out that support from friends, family, and other cancer patients, as well as access to information on illness and diagnosis, may increase the quality of life of cancer patients [21,22].

Unfortunately, many apps have not been evaluated by AYAs through processes including usability testing [23,24], which may affect the app’s quality and appeal for its intended target audience [25]. Additionally, experts in the field seldom evaluate health apps according to the quality and validity of the provided information, which could potentially endanger patient safety [11,23]. This study seeks to address these concerns by investigating the results of a usability think-aloud test of an app, which was created on the basis of initiative and ideas from AYAs with cancer, developed in a co-creation process with an eHealth solution company, and validated by experts. The app is intended to strengthen the quality of life and empowerment in AYAs with cancer [26]

The aim of this study was to explore how the app Kræftværket was used by AYAs who were either on treatment for cancer or in follow-up as well as to investigate the strengths and weaknesses of the app. The results from this study will contribute to the improvement of the Kræftværket app, so that it may serve as a tool that Danish AYA cancer patients will benefit from in the future. Additionally, the study may inspire the development of future apps in other countries aimed at AYAs with cancer.

## Methods

### The Kræftværket App

This study is based on a project located at Kræftværket, a youth support center and social organization for AYAs (aged 15-29 years) with cancer at the Copenhagen University Hospital Rigshospitalet in Denmark. The name of both this center and the mobile phone app described in this study—Kræftværket—is composed of the Danish words for “cancer” (kræft) and “power plant” (kraftværk), evoking empowerment throughout the time of cancer treatment and recovery. The idea of creating an app arose from AYAs at Kræftværket, who saw the need for a tool to strengthen the quality of life and empowerment in AYAs with cancer. Therefore, health professionals from Kræftværket decided to host a series of workshops in which current and former AYA cancer patients could assist in the development of the app in a co-creation process partnered with an eHealth solution company. The app development process was divided into three phases, with this study describing the usability testing of the prototype app as part of phase II (Table 1). The co-creation process is described in its entirety in Elsbernd et al (2018b) [27], and the three phases are described in detail in Elsbernd et al (2018a) [28].

**Figure 1:**
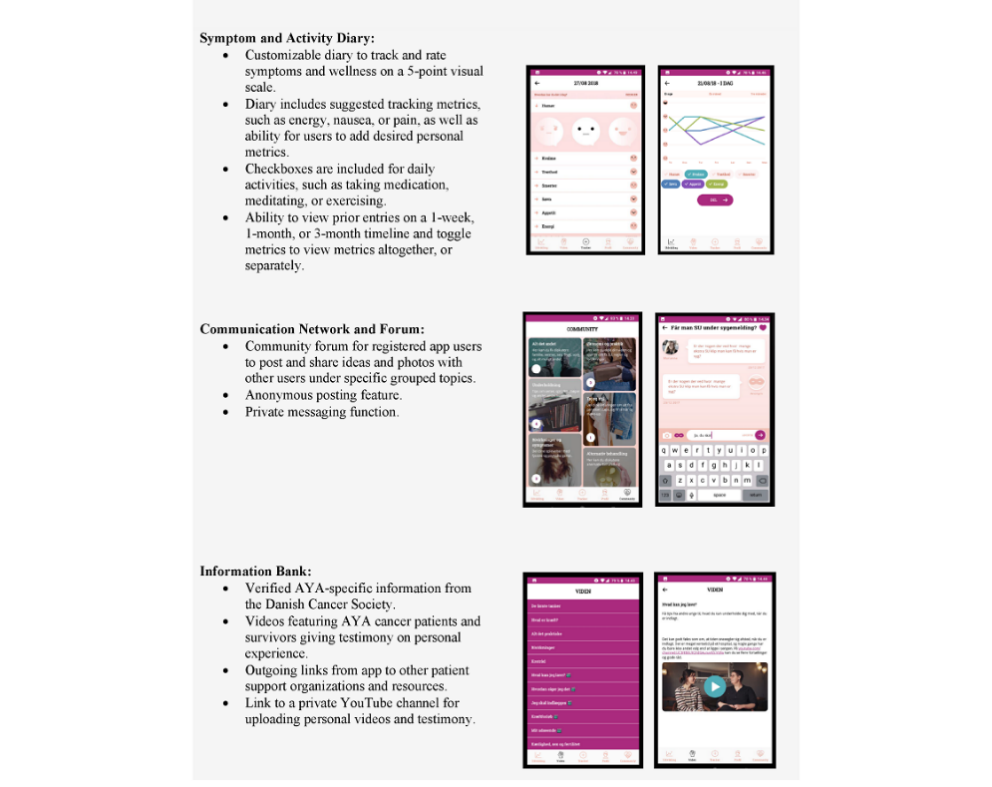
Version 1 content and design of the Kræftværket app. Text from Elsbernd et al [<xref ref-type="bibr" rid="ref28">28</xref>].

The first version of the Kræftværket app was ready for usability testing in April 2018. It contained a symptom and activity diary, a communication network and forum, and an information bank (presented in Figure 1). The app’s community forum was intended to connect and build a community for AYAs with cancer, and the symptom tracker was intended to strengthen the understanding and management of the illness and its symptoms. The app would additionally serve as an information bank with relevant information about cancer diagnosis and implications on youth life. Lastly, the app may serve as a tool for collecting data and knowledge for research.

**Table 1:** Kræftværket app development phases.

Phases, content			Participants, N
**Phase I: initial work (2017/18)**			17
	Literature review and research protocol [28]		
	Initial technology workshop		
	Co-creation workshop and ad hoc meetings [27]		
**Phase II: pilot-testing the prototype (2018/19)**			20
	EORTC QLQ-C30 [26]		
	Think-aloud test^a^		
	Focus group interviews		
**Phase III: implementing and testing the final app (2020)**			50
	EORTC QLQ-C30		
	Focus group interviews (funding achieved)		

^a^Current study.

### Participants and Recruitment

The participants for phase II—pilot-testing of the app—were recruited in the youth support center Kræftværket by a youth coordinator (MH). The pilot testing involved participation in usability testing via think-aloud testing, focus group interviews, and health-related quality of life questionnaires, such as the European Organization for Research and Treatment of Cancer quality of life questionnaire (EORTC QLQ-C30; Table 1). The results of the EORTC QLQ-C30 health-related quality of life testing are described in Taarnhøj et al [26]. The results from the focus group interviews will be published separately. Phase II inclusion criteria were users of Kræftværket, AYAs between 15 and 29 years of age with access to a mobile phone and the internet, including cellular data or Wi-Fi. Exclusion criteria were AYAs who had participated in the co-creation process for the development of the app, and those unable to read and write in Danish. A total of 20 AYAs were recruited: 10 AYA currently receiving cancer treatment and 10 AYA who had completed cancer treatment and were in follow-up. In total, 15 AYA participated in the think-aloud test because five persons dropped out for various reasons, including acute illness on the day of the test (Table 2).

**Table 2:** Demographic and clinical information of participants.

Demographic		All participants (N=20)	Participants who attended think-aloud test (n=15)	Participants who dropped out (did not attend think-aloud test) (n=5)
**Gender, n**				
	Male	6	4	2
	Female	14	11	3
Age (years), mean (range)		25 (16-29)	25 (16-29)	21 (18-29)
**Treatment, n**				
	On treatment	10	8	2
	Off treatment	10	7	3
**Cancer type, n**				
	Lymphoma	9	6	3
	Breast	4	3	1
	Head and Neck	2	2	0
	Leukemia	1	1	0
	Testicular	1	1	0
	Ventricular	1	1	0
	Thyroid	1	1	0
	Brain	1	0	1

### Setting

The usability test consisted of a 6-week test period (April-May 2018) in which the participants were given access to the app prototype. They were instructed to use the app according to their needs; however, they were not given any specific instructions on the frequency with which to use the app. After the test period, the participants arrived and were asked to participate in the think-aloud test, which was administered by the eHealth solution company associated with the co-creation process. The test took place in the youth support center Kræftværket at Copenhagen University Hospital, Rigshospitalet, in May 2018. The test was performed individually with one participant at a time, and each test lasted 10 minutes on average (range 7-13 minutes). All tests were video-recorded.

### Think-Aloud Test

Usability was evaluated using the think-aloud method to test the app’s functionalities as well as opinions, comments, and concerns among users [29]. The think-aloud test provided an overview of which functionalities (eg, understanding navigating paths and use of buttons) were causing the greatest difficulties and which functionalities were best received by the users. The test assessed how participants perceived icons, menus, and navigation paths. As part of the evaluation, participants were given minor tasks using the app, which the participants were asked to complete while describing their thought processes aloud along the way with questions such as:

- What do you think you can do on this page?
- How do you track your side effects?
- How do you do to add a symptom to the tracking feature?
- How do you find the community?
- How do you write an answer to a question in the community?
- How do you upload a new profile picture?

SH performed the transcriptions of the think-aloud tests. Verbal statements were transcribed verbatim with physical actions incorporated into the transcript and identified with square brackets ([]), which are included in the quotations. All physical actions were included in the transcript, including the exact functionalities or features of the app that were used when the participants scrolled, pressed buttons, took pictures, and more.

### Data Analysis

Data were analyzed using a thematic analysis approach inspired by Braun and Clarke’s 6-step model [30]. Coding of text involved reading and re-reading the transcriptions to identify and categorize concepts across data relevant to the research question. Concepts were highlighted in the margin of the transcriptions. The authors SH, MH, KAB, and HP completed this initial coding separately to ensure an independent coding process. They subsequently met to discuss and sort the concepts into themes and subthemes, and each author presented their coding. In a joint process, the researchers came up with suggestions for themes and subthemes, which were written on sticky notes and placed on a table to create a good overview. There was an immediate agreement on themes; subthemes were arranged and rearranged during constructive discussions until agreement was reached.

### Ethical Considerations

The study was approved by the Danish Data Protection Agency (VD-2018-27). Ethical approval is not necessary for this kind of study in Denmark; however, the principles stated in the Helsinki II Declaration were followed. All informants received oral and written information before the think-aloud test and were provided written informed consent to participate. All informants were given the opportunity to withdraw their consent without any consequences for the treatment at the hospital. The video-recordings consisted solely of recordings of the participants’ hands and mobile phones. Participants’ faces and other identifying characteristics were not recorded.

## Results

### Data Analysis

Data analysis yielded results on how the app was used, including the relation to gender and treatment status, as well as strengths and weaknesses of the prototype app and suggestions for improvements. The following themes were identified: navigation, visual and graphic design, and usefulness (Textbox 1).

**Themes and subthemes consisting of strengths, weaknesses, and suggestions for improvements.**

1. Navigation
  - Intuition
  - Features
  - Buttons
  - Home page
  - Profile
2. Visual and graphic design
  - Overview
  - Text and colors
  - Photos
  - Videos
  - YouTube
3. Usefulness
  - Notifications
  - Posts
  - Adding

### Navigation

#### Intuition

Many of the participants assessed navigation paths and whether they were intuitive in nature. Navigation was determined to be intuitive if it imitated the navigation paths used in other media, such as existing apps and text message systems, and if it seemed clear, simple, and user-friendly: “It is very simple, it seems very intuitive, it works very similar to other media” (female participant). For example, the buttons in the symptom and activity diary, liking conversations in the community with hearts, and the creation of conversation threads in the community were perceived to be intuitive.

#### Features

##### Tracking and Scale

A key feature of the app was a symptom and activity diary with patient-provided tracking features. It consisted of a scale containing five smileys to track the following symptoms: mood, nausea, fatigue, pain, sleep, appetite, and energy. Additionally, it included an activity diary to record activities such as running, cycling, yoga, social activities, and alcohol consumption. After tracking, it was possible to view the statistics over time for periods of one week, one month, and three months. Tracking of symptoms was the feature most frequently used during the test period, and most participants were very excited about it. It helped them remember daily events and symptoms, which one participant noticed could be a challenge during chemotherapy. Additionally, it could be referred to when visiting the doctor, and it provided a good overview of symptom progression. The participants found it easy and straightforward to use. However, the think-aloud test led to some suggestions for changes. When choosing a symptom, it was not possible to proceed without first rating the symptom, although it might not be relevant at all. Also, several of the participants had difficulty interpreting the smileys. For example, they did not know if “being very nauseous” should be rated with a happy or a dissatisfied smiley. Some participants suggested replacing the smileys with a number:

I don’t know if I’m so much a supporter of smileys. Perhaps I’m more a supporter of numbers, because now I’m suffering from a lot of headaches [presses on “headaches”], and I have chosen to say if I don’t have a headache, then it is a happy smiley [presses on the happiest smiley], and there I would have preferred numbers instead.

Female participant

Not all participants were able to find the first and last smiley on the scale because they did not notice they had to scroll sideways to see them. Also, it was suggested to place the tracker and statistics next to each other in the menu because they were linked features. Notably, many of the participants stated that using the tracker was most relevant during their treatment course, and the tracker was perceived to be the main reason to use the app; as such, the app was determined to be a stronger tool for patients in treatment rather than after treatment.

##### Notes

In the symptom and activity diary, it was possible to write notes for a specific day. The user was encouraged to write a note with the following app-provided teaser: “What have you been particularly aware of? Write here.” Most participants thought it was a good idea to make room for notes because they had the opportunity to elaborate on why they might be particularly tired one day or what made a given day particularly good. Unfortunately, most of the participants were unable to find old notes because old notes could only be found by clicking back to the day they were written: “It was a very long time ago, I don’t know when I wrote it [scrolls up and down and press the back button (the arrow) at the top—repeats this action 5-6 times but finds no notes]” (female participant). Because of this, one could risk having to click 30 times back if notes were written one month ago. One participant suggested adding notes as an element of the statistics, which remained constant. This could then increase the overview and minimize clicks.

#### Buttons

##### Edit

In the symptom and activity diary, it was possible to press the button to edit and add a new symptom or a new activity to the tracker. Many of the participants were happy about adding new features because it could tailor the app to their individual needs during their cancer course. The participants did, among other things, add Kræftværket (number of visits), menstruation, fertility, sexual health, bloating, and headache to the symptom tracker and walks, work, studies, and treatment dates to the activity tracker. During the think-aloud test, it became clear that the female participants were more familiar with the edit button than the male participants, as only women had added new symptoms and activities during the six-week test period. None of the male participants had noticed the edit button for adding new activities. One male participant explained that he had misinterpreted the wording of edit: “I haven’t seen it before, but now I can see that I can even add...I wouldn’t call it ‘edit,’ something with ‘add’ maybe” (male participant).

##### Back Arrow

Several of the participants had difficulty using and figuring out the use of the back arrow button, designed to return to the previous page. The main problem was that previously entered data disappeared when the arrow was pressed: “I have repeatedly pressed the arrow up here, because I wanted to return [pointing to the arrow in the upper left corner], because everywhere else this is a back function, but then it erases it all, and then I can start over” (male participant). Some suggested inserting a save button, which could be used when new information had been entered (eg, in the tracker). This could increase the feeling of security with data entered because many were in doubt about what would happen to their data when they pressed the arrow.

##### Share

In the symptom and activity diary, it was possible to see one week, one month, or three months’ timeline and toggle metrics to view statistics. Under the statistics, there was a button called “share.” When pressing the share button, it was possible to attach the statistics to a text message or an email by selecting from the phone book. Some participants were not concerned with sharing their statistics and could see an advantage to sharing with others, such as a doctor or a nurse, with prior arrangement. However, most participants were unsure of who to share the statistics with: “I can’t quite imagine who to share it with, if I have to be honest” (female participant). A single participant did not choose to touch the button at all because he was afraid and unsure of who would receive the statistics when sharing. He did not know that the button was associated with his own address book.

##### Icons

The participants evaluated the following icon buttons: Anonymous (a masked face), Knowledge (a clenched fist), Videos (a clapboard), Upload Photo (camera), and Upload Text (arrow). The participants found the icons to be user-friendly, and most participants recognized them from other social media, such as Messenger, which aided in their understanding. The only icon they could not associate with its contents was the clenched fist—the icon for knowledge—which was used to link to the information bank. One suggested a book icon instead: “It is a very nice icon and a little like, ‘We stand together,’ that is good, but I don’t know if it is so much related to knowledge, I might have thought of a book or something like that” (female participant).

### Home Page

The symptom and activity diary served as the home page when opening the app, with the mood tab open and available for ranking. Three smileys were visible under mood: a sad smiley, a dissatisfied smiley, and a happy smiley. Some participants thought it was a bad idea to be met by an open tracker if they did not intend to use the tracker on that given day, whereas others thought it was inconvenient to be met by non-happy smileys: ”When you go into it you just see some smileys, and the first is the ‘I’m not so happy.’ Here I just really think it should be closed” (female participant). More participants called for a visible home page with an overview of the app content to get a better insight regarding the content and an idea of how to navigate.

#### Profile

The profile consisted of a place for users to provide and manage their personal app information, including a photo, name, city, email, password, day of birth, cancer type, and gender. Additionally, a logout button was provided. Most profile settings were optional except for name, email, and password. Several participants were doubtful about what the profile should be used for and why it was there at all. More participants were concerned about whether others could see their profile settings because these were private. Conversely, one participant thought it was smart to have the opportunity to see other app user’s profile information (eg, city). With that information, it would be possible to contact others to meet in real life. The password was marked with 18 dots, which confused all participants who were asked to recall their password and reprint it. No one could remember if they had ever logged in with a password, and found it confusing that the number of dots did not match the number of characters in the correct password: “It looks like I have a really long password—[clicks on it and it doesn’t respond] I don’t think I have, and I can’t change it either” (female participant). It was also possible to change the day of birth in the profile, which one participant questioned because this never changes. Additionally, it was possible to choose a day of birth later than today’s date.

### Visual and Graphic Design

#### Overview

Some participants called for a better overview of the information bank, the tracking statistics, and community threads. Additionally, some participants lost the overview of the subpages in the information bank, as all the headings were named “knowledge,” which did not reflect the content of each page. However, it was noted that the order of information in the information bank followed the chronology of a cancer course, which was considered an advantage: “I think the topics make really good sense, also the sequence they follow, because it is usually in the order that things actually happen” (female participant). A few participants lost the overview in the tracker when all symptoms were shown simultaneously in the statistics. However, most participants thought the statistics were easy to understand because each symptom had its own color in the curve and could be clicked on and off as needed. Moreover, some participants found it difficult to get an overview of replies in the community, especially if there were many replies in the same thread, as they appeared in one long vertical row requiring scrolling down the page.

#### Text and Colors

Most participants were satisfied with the amount of text on the pages, even though opinions differed whether the information bank was too text-heavy or not. However, the information bank was welcomed by all participants because it could replace the many paper flyers the AYAs had received at the time of diagnosis and it was targeted toward AYA, unlike some of the flyers and handouts. The participants additionally found that the colors in the app supported the features: “I think the color coordination makes sense” (female participant). One participant noted that the color scale was very pink and a little too distracting.

#### Photos

It was possible to upload photos in two different places in the app: profile photos and photos for posts in the community. The participants found it easy and straightforward to upload photos, but it was considered a problem that profile photos could not be deleted: “I have no desire to have a picture in there, so now I have problems, I think” (female participant). When a profile picture was uploaded, it was not possible to remove it, but only to replace it with another photo. This was concerning for those who wanted to remain anonymous.

#### Videos

In the app’s information bank, approximately half the topics were accompanied by a short video, in which AYAs from Kræftværket spoke about their personal experiences with a given topic. Several participants thought the videos were easy to find and play. Additionally, they were excited about the videos because it was nice to identify with real people and it increased the feeling of normalcy during the cancer course. It was suggested to link videos with all the available information bank topics: “It is a pity that there are not videos on all of them actually...It could be nice if there was also a face on...So that you can think okay, I’m not completely abnormal” (female participant).

#### YouTube

In the app’s information bank there was a link to a private YouTube channel for uploading personal videos and testimonies. Two participants were asked about their use of YouTube, but they had not found this feature during the test period. One of the participants thought it was difficult and confusing to upload videos, and the other participant thought that YouTube was an inappropriate medium for the users of the app and she could not imagine speaking about herself and her cancer course on YouTube because it was too personal: “For me Kræftværket does not belong to YouTube, there are some other platforms...I can’t even see myself using it” (female participant).

### Usefulness

#### Notifications

Most participants missed notifications because they were not getting reminders of new posts in the community, and they did not know whether their own posts were commented on by others. As a consequence, they often forgot to use the app. They pointed out that if the app was to become a useful tool to them, notifications should work to serve as a reminder: “Notifications, I really, REALLY think are missing. It’s probably 70% of the reason why I do not use this app” (female participant). Furthermore, notifications could make the app more dynamic as they would create visible activity.

#### Posts

In the community, there were several problems with posting, which had an impact on the credibility and desire to use the app. First, the participants pointed out that the number of posts were not counted correctly on the community page, so the actual number of posts did not match the visible number: “My posts don’t pop up on the front page [the speech bubbles show ‘0’ on the front of the community despite of her post]” (female participant). In addition, it was unclear whether the system counted every single post or the number of responses related to each post. It was possible to give likes to posts using a heart icon, but it was unclear whether likes were given to a full conversation thread or only a single post. Moreover, dates for each individual post were missing, so it was impossible to distinguish between new and old ones. It was mentioned that posting in the community was mostly relevant during the cancer treatment period where concerns and questions about issues related to life with cancer were urgent.

#### Adding

Several participants mentioned that the app could work better by connecting with other apps and media, so the users did not have to switch between many different apps. It was suggested to link to relevant Facebook pages, the Epic system (electronic health records), the Peak app (training of cognitive skills), and health apps (eg, running and cycling): “It could be smart if you could get something to pull data from your health app because...I forget (the Kræftværket app) a little” (male participant). It was additionally suggested that the app should register attendance in the youth support center Kræftværket. The app could also be made more dynamic by having the opportunity to add new main topics to the community as opposed to using preset topics, which would further customize the app to the needs of the individual.

## Discussion

### Principal Findings

This qualitative study of a think-aloud test highlighted the strengths and weaknesses of a prototype of a mHealth app aimed at AYAs during and after cancer treatment. The study is among a limited number of usability studies evaluating health apps [31]. Additionally, there is a lack of studies that actively involve cancer patients, including AYAs, in usability testing of apps, which this study seeks to address [24,32]. Many studies focus on testing the outcome and effectiveness of cancer apps in terms of self-management and adherence to treatment [12,33,34]. These studies are highly beneficial and are necessary to understand the utility of these apps; however, lack of usability testing may lead to the development of unsuitable navigation paths as well as content and design that does not appeal to AYA with cancer, which may limit their use.

The results of our study discuss two of the main functionalities in the app—the tracker and the information bank—which were perceived as strengths by most participants. AYAs were particularly excited about the app’s tracking function, where they could track activities and symptoms such as mood, energy, and sleep, and get statistics on the tracking for one week, one month, and three months. The tracking was found to be a strong tool for recalling activities, events, moods, and symptoms, which could be used at hospital visits or consultations. This tool could also be tailored to the individual needs of AYAs during treatment. In line with this, research has shown that personalization of apps may better adjust to the needs of young adults, which provides a positive user experience [35]. Adolescents often perceive tracking of personal health data as a benefit [36]. However, in contrast to a study on adolescents with asthma who welcomed sharing health data with email recipients [37], the participants in our study were generally more skeptical about sharing health data, and more were in doubt about who they should even share data with.

In our study, some participants suggested using a numerical scale to rank symptoms instead of using smileys. This is in line with suggestions raised by adolescents with cancer testing a pain app [32]. The information bank was perceived as a strong reference tool for seeking information during the cancer treatment course. Topics containing short videos were especially well received because it was possible to identify with the stories of other AYAs, which increased the feeling of normalcy. The use of videos has also been perceived as useful in other app-testing studies [38]. Video cancer narratives have demonstrated a significant therapeutic effect for those AYA who create the narrative [39], but few studies describe the effect of watching other’s cancer video narratives. However, studies on narrative communication in adult cancer prevention and control point to the importance of parasocial connections between the storyteller and the audience [40], because authentic narratives impact recipients emotionally and thus both benefit and inspire the audience [41].

As found in other studies, the participants in our study wanted a clear, concise presentation of information, which should not be too text-heavy [35,42]. The think-aloud test also led to several suggestions for improvement of the app, which should be taken into consideration when developing apps for AYA cancer patients. For improved navigation, the wording and design of buttons and icons should be very precise for correct understanding. As such, the wording of “edit” was ambiguous, and it was not clear what happened when using the back arrow button. Moreover, some had difficulties scrolling sideways. Difficulty in managing navigation levels, including relying on the back button as a safe option, as well as difficulty in scrolling and performing swipe gestures have been found on app usability [35,43,44]. Additionally, an overview of content should be very clear. A suggestion to accomplish this was the creation of a home page presenting all content of the app, which is in line with the idea to have a dashboard that brings together all the features that the app provides [45].

We found that anonymity should be a very high priority; as such, it should be possible to delete uploaded photos, and the app should be transparent about who personal information was available to. In contrast to our study, other research has found that young people with asthma were interested in allowing the upload of pictures to make the profile more interactive, with less concern regarding anonymity [46]. In line with the need for anonymity, we found that YouTube may not be a relevant media in relation to the app, as information regarding illness and cancer is a private matter for some AYAs. As such, not all AYAs were interested in telling about their illness publicly. The linked YouTube channel was set to be private, but it seemed this was not sufficiently clarified because the participant who reflected on it found it too personal to post a video. The need for privacy contrasts with studies on adult cancer survivors using YouTube for their personal narratives [47,48]. Reasons for posting personal videos on YouTube were, for example, to provide support and advice for others in the same situation and raise awareness of aspects of the diagnosis [48]. Keeping illness a private matter is a general theme among adolescents with chronic illness. Illness is often associated with abnormality and being old and dependent on others, in contrast to the normal, healthy identities AYAs wish to create [49]. Visible body changes, which are known side effects of cancer treatment, highly affect body image and identity formation [50] and may be another detractor to the use of YouTube in this app.

Notifications were found to be one of the most necessary features for participants to use the app at all. The app did not contain a notification system; therefore, the participants missed reminders when their posts were commented on by others, which made the app static and less user-friendly. Also, problems with posting in the community were associated with reduced use of the app; it was suggested that it had to be more transparent how posts were counted in the community. A better understanding of the ordering and interaction on posts could make the app more dynamic and engaging. Previous research on adolescents’ use of apps has shown that reminders could improve usability [35]. In line with other research, we found that the Kræftværket app was perceived to be most relevant during a cancer course in which most AYA lacked knowledge about diagnosis and treatment. Tracking of symptoms, information seeking, and asking other AYAs for advice seem to be most relevant at diagnosis onset [6]. In our study, we also found some gender differences in the use of tracking as few male participants had used the edit button in tracking of symptoms. One male misunderstood the wording of “edit,” and none of the young men had noticed the possibility of adding new activities in the activity tracker. Existing research confirms gender differences in the usability testing of apps as female cancer patients have shown to be more persistent test users by using apps continuously and generally more frequently during test periods than males [51,52].

### Limitations

Some limitations should be taken into consideration. The duration of the think-aloud test was only approximately 10 minutes per person; therefore, the participants were each assigned different tasks and questions to uncover all aspects of the app. As a result, some of the app’s functionalities were tested more thoroughly than others, which may be reflected in the emphasis of various themes in the study. The app was developed for two target groups: AYAs during and after active treatment. Approximately half the test users noticed that the app was primarily relevant during cancer treatment, which is an aspect that earns more attention in the forthcoming evaluation of the app. At present, it is not known how the app will be used by AYAs who get access to it during treatment and then continue using it during follow-up. This study highlighted how a cancer app, which was developed in a co-creation process involving AYAs, was received by other AYA test users. Even though the app was developed in a co-creation process, the test users had several suggestions for improvement, reflecting that AYAs have individual needs and opinions about apps that cannot all be met.

### Conclusion

The think-aloud test of the Kræftværket app has led to some conclusions relevant for the general development of youth-specific cancer apps. Usability testing is crucial during the app development process to improve the app according to the needs of the target audience. Additionally, cancer apps aimed at AYAs should contain relevant, targeted, and unique content, preferably including a symptom and activity tracker and videos presenting relevant advice from other AYAs with cancer experience. Notifications are necessary to remind AYAs to use the app, and ordering and interaction of the posts should be transparent and precise to satisfactorily create a dynamic and engaging app. Further research is recommended to evaluate the Kræftværket app with the input of more AYAs in other hospital settings and for a longer test period covering the trajectory of the participant’s illness and recovery.

## Abbreviations

AYA: adolescent and young adult
